# Advances of hafnium based nanomaterials for cancer theranostics

**DOI:** 10.3389/fchem.2023.1283924

**Published:** 2023-11-24

**Authors:** Jiayi Wang, Jiahua Pan, Yijun Tang, Jingqi Chen, Xiaochen Fei, Wei Xue, Xueliang Liu

**Affiliations:** ^1^ Department of Urology and Institute of Molecular Medicine (IMM), Renji Hospital, School of Medicine, Shanghai Jiao Tong University, Shanghai, China; ^2^ Department of Endocrinology and Metabolism, Shanghai Children’s Medical Center, School of Medicine, Shanghai Jiao Tong University, Shanghai, China

**Keywords:** Hf-based nanomaterials (Hf-NMs), bioimaging, cancer therapy, combination therapy, biosafety

## Abstract

Hafnium-based nanomaterials (Hf-NMs) have attracted the interest of numerous biomedical researchers by their unique properties. Recent years have witnessed significant advancements in the field of Hafnium-based nanomaterials, particularly in the context of cancer diagnosis and treatment. However, research in this area, especially concerning the clinical application of Hafnium-based nanomaterials, has not been thoroughly reviewed. This review will cover: 1) Classification and synthesis of Hafnium-based nanomaterials including Hafnium oxide nanomaterials, Hafnium Metal-Organic Frameworks/nanoscale coordination polymers (MOFs/NCPs); 2) Hafnium-based nanomaterials act as contrast enhancement agent for cancer imaging, and hafnium-based nanomaterials used for diagnosis in cancer liquid biopsy; 3) hafnium-based nanomaterials for cancer therapy, including hafnium-based nanomaterials for radiotherapy, hafnium-based nanomaterials for photodynamic therapy, hafnium-based nanomaterials for various combined therapy; and 4) Translation, toxicity, and safety for Hf-NMs in human and preclinical animal models. More attention will be given to the clinical translation of Hf-NMs in cancer.

## 1 Introduction

Cancer has become one of the most common diseases threatening human life and health all over the world (Siegel et al., 2022). Specifically, individuals diagnosed with cancer at advanced stages often face suboptimal clinical outcomes, emphasizing the critical need for innovative strategies focused on early detection and personalized treatment. In the past decade, the burgeoning field of nanomedicine has taken significant steps forward in developing diagnostic and therapeutic agents for a variety of diseases, with a series of nano-systems being actively explored (Wang et al., 2021). In this multi-disciplinary field, multifunctional NMs are of great concern as they are strongly associated with diagnostic performance and therapeutic efficacy, which is a major focus of nanomedicine research (Dong et al., 2020; Yang et al., 2021; Chen et al., 2022). Especially, the last decade has witnessed remarkable progress in the synthesis process and therapeutic application of the inorganic NMs in cancer researches (Wang et al., 2021).

Studies have demonstrated that nanosystems containing high atomic number elements have been widely used as radiosensitive agents and biomedical imaging due to their inherent X-ray attenuation coefficient. They can effectively absorb high-energy rays and generate cytotoxic free radicals, enhancing therapeutic effects. Hafnium (Z = 72, Hf), a chemically stable metallic with high atomic number and electron density, stands out as one of the frequently employed high Z inorganic NMs (Ushakov et al., 2019). Many studies have explored the application of Hf-NMs in cancer theranostics, so it is necessary to classify and review Hf-NMs from various perspectives. There are many different types of Hf-NMs, mainly including Hf oxide NMs, Hafnium Metal-Organic Frameworks/nanoscale coordination polymers (MOFs/NCPs), and Hf carbon dots. These Hf-NMs are synthesized using diverse methods such as sol-gel, hydrothermal, and wet chemical precipitation, providing them with a range of morphologies and functions that are increasingly influential in cancer theranostics (Dominguez et al., 2017). In this review, we categorize Hf-NMs and present their principal synthesis methods.

The different properties of Hf-NMs can help in cancer imaging and diagnosis. Due to previous studies demonstrating their excellent mass attenuation coefficient and ability to accumulate in tumor tissue and enhance irradiation (Martínez-Rovira and Prezadoa, 2011), Hf-based NMs can serve as valuable X-ray and CT imaging contrast agents, and have been extensively investigated as multimodal bioimaging contrast agents (Mendoza et al., 2010; Pottier et al., 2014; Frenzel et al., 2016; McGinnity et al., 2016). Additionally, Hf-based nanosensors have the capacity to aid in tumor diagnosis by detecting cytokines in various body fluids. This section of the review emphasizes the utilization of Hf-based NMs as contrast agents in cancer imaging and as nanosensors in cancer liquid biopsy.

In cancer treatment, Hf-based NMs have been employed in clinical settings to enhance radiotherapy. One representative Hf-based NMs, NBTXR3, has shown promising results as a radiosensitizer in human clinical trials, achieving effective tumor control. Besides, studies have reported that Hf-based NMs can achieve X-ray-induced photodynamic therapy (X-PDT), which can absorb the high energy of radiated photons to treat deep tumors. Preclinical studies also highlight the considerable treatment efficacy of Hf-based NMs when combined with conventional therapies such as photothermal therapy (PTT), chemotherapy, and immunotherapy (Pottier et al., 2014). This review consolidates the utilization of Hf-based NMs in enhancing radiotherapy and X-PDT in monotherapy. Additionally, it provides a summary of combination therapies, including radiotherapy coupled with PTT, chemotherapy, and immunotherapy.

In the clinical translation of nanomaterials, addressing safety and toxicity is paramount. Current research indicates that Hf-based NMs exhibit a favorable safety profile with manageable toxicity (Field et al., 2011; McGinnity et al., 2021). Extensive investigations into Hf-NMs have been conducted in the realm of cancer therapy, spanning preclinical animal models to clinical translational applications. Consequently, it is crucial to comprehensively outline the safety and toxicity of Hf-NMs in current studies, offering valuable insights for future clinical translation. This review marks the inaugural summary of clinical studies on Hf-based NMs, delving into a detailed discussion on their safety and toxicity. Additionally, the review provides prospective insights into potential clinical scenarios for future applications.

**SCHEME 1:**
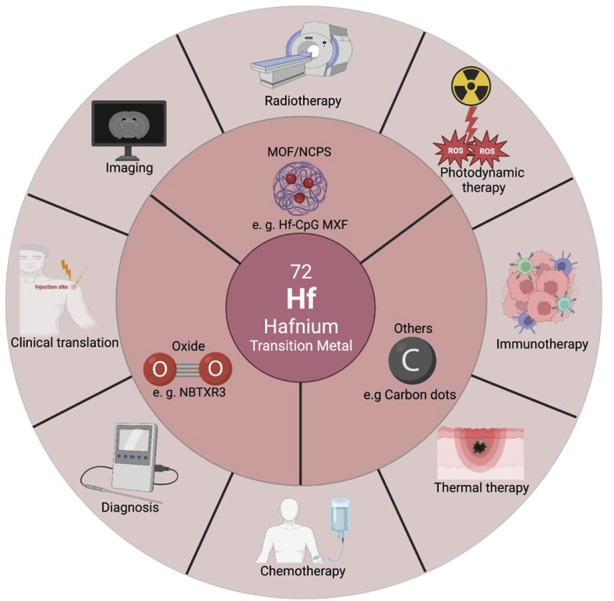
Illustration of classification, imaging and diagnosis, cancer mono/multi therapy, and clinical translation of Hf-based nanomaterials.

Nevertheless, oncologists and chemists typically possess specialized knowledge within their respective domains, underscoring the significance of a comprehensive multi-disciplinary summary of diverse treatment approaches. In brief, it is urgent and necessary to systematically summarize the recent progress of Hf-NMs in bioimaging and cancer theranostics. Presently, existing reviews predominantly concentrate on preclinical studies using animal models. In this review, we introduce the synthesis of different types of Hf-NMs, including HfO_2_ NMs, Hf-based MOFs/NCPs, Hf carbon dots, *etc.*, and their capability to largely improve cancer imaging and diagnosis effectiveness. Next, we introduce the application of Hf-NMs in cancer monotherapy, such as radiotherapy and photodynamic therapy. Moreover, we highlight the additional benefit of combined therapies. Drawing from published reviews, we comprehensively summarize ongoing clinical trials involving Hf-based NMs. Furthermore, we address the toxicity and biosafety considerations associated with the biomedical applications of Hf-NMs, discussing progress in the translational process, encountered challenges, and potential future clinical scenarios (Scheme. 1). Additionally, we compile the latest published literature (Chen et al., 2023; Ding et al., 2023). Our aspiration is that this review can serve as a guiding framework for future researchers using Hf-based NMs in advancing cancer diagnosis and therapy.

## 2 Hafnium based materials

### 2.1 Hf oxide NMs

HfO_2_ exhibits remarkable chemical inertness, a high dielectric constant, elevated melting point, density, refraction index, and visible light transparency. These properties, coupled with its minimal reactivity in biological systems, position HfO_2_ NMs as promising candidates for roles as radiosensitizers and X-ray contrast agents. For instance, HfO_2_ NMs as radio-enhancers will generate a large number of electrons under X-ray irradiation, thereby promote the killing ability of cancer cells (Mendoza et al., 2010; Maggiorella et al., 2012).

There are two main synthesis methods for HfO_2_ NMs, sol-gel method and hydrothermal method. The size and morphology of NMs were adjusted during synthesis by changing temperature, time, pH, reactant ratio and other factors. McGinnity designed HfO_2_ NPs as an X-ray contrast agent and mid-infrared biosensor via a meticulous sol-gel methodology (McGinnity et al., 2016). The hydrodynamic diameter of the prepared HfO_2_ NP calcined at 575°C was 225 ± 74 nm, which reflected the presence of multi-particle aggregates. Subsequently, their dispersion was achieved through surface functionalization employing polyvinylpyrrolidone (PVP), followed by a process of ultrasonication. The hydrodynamic diameter measured by DLS for PVP-HfO_2_ NPs calcined at 575°C was 133 ± 74 nm, transmission electron microscopy (TEM) micrographs showed that PVP-HfO_2_ NPs were spherical and well-dispersed. Similar methods for the preparation of HfO_2_ NPs have been used in several studies.

Besides sol-gel method, Li, et al. reported the synthesis of biocompatible and intravenously injectable HfO_2_ nanocrystal assemblies (NAs) synthesized by microwave-hydrothermal (MH) method (Li et al., 2020). HfCl_4_ was stirred at 80°C for 1 h, after which the sodium hydroxide solution was slowly added and stirred for 30 min at room temperature. Then the mixture solution was sealed and placed inside the MH system, MH conditions were set at 160°C for 1 h. The resulting solution in the Teflon autoclave was then centrifuged to collect the white colloidal precipitate. CT imaging-guided radiotherapy sensitization of cancer was achieved by synthesized HfO_2_ NAs.

NBTXR3, the most used hafnium oxide NPs till now, was developed by the French company Nanobiotix. These NPs are coated with a biocompatible agent that imparts a negative surface charge, which ensures their stability in aqueous solutions within the pH range of 6–8. They have a size of 50 nm and polydispersity index of about 0.1, zeta potential about −50 mv^17^. The synthesis method of reported NBTXR3 NPs, which composed of crystalline HfO_2_ covered by a negatively charged phosphate coating, has not been carefully discussed. Also, the morphological structure characterization and modification have not been fully studied. In functional experiments, it has been proved that NBTXR3 can significantly enhance the efficacy of radiotherapy *in vivo* and *in vitro*, with controllable biological toxicity (Marill et al., 2014; Bonvalot et al., 2017; Bonvalot et al., 2019; Hoffmann et al., 2021).

### 2.2 Hafnium metal-organic frameworks/nanoscale coordination polymers (MOFs/NCPs)

Metal-organic frameworks (MOFs) and nanoscale coordination polymers (NCPs) are crystalline porous materials formed by self-assembly of organic ligands and metal ions driven by coordination bonds. These materials find diverse applications in nanomedicine, encompassing biosensors, molecular imaging, and therapeutic interventions (Danowski et al., 2019; Feng et al., 2019; Mohammad-Pour et al., 2019; Sun Y. et al., 2020; Hanikel et al., 2020; Skorupskii et al., 2020; Wang et al., 2020). Benefiting from targeted modification capabilities, favorable biocompatibility, and chemical stability, MOFs/NCPs have emerged as viable tools for investigating novel approaches to tumor treatment (Yang et al., 2017a; Cheng et al., 2020; Liu et al., 2021a). The integration of MOFs/NCPs with different materials has yielded innovative composites/mixtures for cancer therapy (Wu and Yang, 2017). Hf-based MOFs/NCPs have become one of the research topics. Below, we introduce Hf-based MOFs/NCPs in cancer diagnosis and treatment (Table 1) (Liu et al., 2016; Liu et al., 2017a; Liu et al., 2017b; Yang et al., 2017b; Chao et al., 2018; Lu et al., 2018; Chen et al., 2019; Gu et al., 2019; Bao et al., 2020; Ni et al., 2020; DuRoss et al., 2021; Li et al., 2021; Sang et al., 2021; Li et al., 2022; Yang et al., 2022; Zhou et al., 2022; Choi et al., 2023).

**TABLE 1:** Hafnium based MOFs/NCPs in cancer diagnosis and treatment.

Hf-based MOFs	Application	Drug-delivery	Structure	References
Hf-CpG MXF	Radiotherapy	intratumorally	MXF	Yang et al. (2022)
UiO-66-NH_2_(Hf)	Radiotherapy	intratumorally	UiO-type MOF	Zhou et al. (2022)
DBP-Hf nMOF	Immunotherapy/Radio‐Radiodynamic Therapy	intratumorally	UiO-type MOF	Lu et al. (2018)
HfMOF-PEG-FA@IMQ	Immunotherapy/radiotherapy	intratumorally	UiO-type MOF	Choi et al. (2023)
Hf-TCPP NMOF	Photodynamic therapy/radiotherapy	intravenously	MOF-535	Liu et al. (2016)
FA-Hf-Mn-NMOF	Photothermal therapy/radiotherapy	intravenously	P6/mmm structure	Bao et al. (2020)
MnTCPP-Hf-FA	Radiotherapy	intravenously	Topology	Chen et al. (2019)
Hf-DBP-Fe	Immunotherapy/radiotherapy	intratumorally	HCP topological structure	Ni et al. (2020)
TT@Hf-BDC-Fuco	Chemoradiotherapy	intravenously	Unformed MOF	DuRoss et al. (2021)
Hf-PSP-DTC@PLX	Radiotherapy	intravenously	Unformed MPN	Li et al. (2022)
CPPDA-Hf@Poloxamer	Photothermal therapy/radiotherapy	intravenously	Core–shell structure MPN	Li et al. (2021)
Hb@Hf‐Ce6	Radio‐Radiodynamic Therapy	intravenously	Unformed MPN	Sang et al. (2021)
Hf-TCPP-PEG	Radioisotope Therapy	intravenously	Unformed NCP	Chao et al. (2018)
NCP-Ce6-DOX-PEG	Chemo-photodynamic therapy	intravenously	Unformed NCP	Liu et al. (2017a)
NCP-PEG/TPPGC	Chemoradiotherapy	intravenously	Unformed NCP	Liu et al. (2017b)
Mn/Hf-IR825@PDA-PEG	Thermo-radiotherapy	intravenously	Others	Yang et al. (2017a)
CDs@ZrHf-MOF	Early diagnosis	—	Others	Gu et al. (2019)

Many relevant studies have focused on synthesizing Hf-based MOFs possessing desirable biostability and optimal particle sizes suitable for administration. The earliest reported work was in 2016, Liu, et al. synthesized Hf-TCPP NMOF NPs. First, HfCl_4_, TCPP and acetic acid were added to a glass bottle, maintained in an oven at 80°C. After 2 h, dimethylformamide (DMF) was added to the reaction system for an additional 24 h. Dark purple solid products were collected by centrifugation and subsequently washed with DMF, triethylamine/ethanol, and ethanol. Finally, the purified NMOF NPs were redispersed in chloroform for further use. Synthesized Hf-TCPP NMOF adopted ftw topology (MOF-535), combined the photosensitizer TCPP with the radiotherapy sensitizer Hf^4+^ to achieve enhanced radiotherapy and PDT (Liu et al., 2016). Later, the same group used polyhistidine-polyethylene glycol (pHis-PEG) copolymer as the stabilizing agent to prepare Hf-TCPP-PEG and found it could be easily labeled with an imaging radioisotope 99mTc. Meanwhile, Hf^4+^ act as a radiosensitizer, Hf-TCPP-PEG can enhance the radioisotope therapy efficacy of 99mTc by absorbing γ rays and emitting charged particles (Chao et al., 2018). Additionally, efforts have also been directed toward enhancing tumor targeting effects by modifying hafnium-based MOFs with folic acid. Bao and Chen, et al. both conjugated the carboxylic acid end of folate based on Hf-Mn-TCPP by coordination, obtained radiotherapy enhancement and PTT (Chen et al., 2019; Bao et al., 2020). Duross, et al. firstly suspended nMOF Hf-BDC with fucoidan or dextran, and then loaded with DNA damage repair inhibitors (temozolomide/talazoparib). The synthesized TT@Hf-BDC-Fuco nMOF exhibited better tumor control, enabling enhanced chemoradiotherapy (DuRoss et al., 2021). In another study, the surface of Hf-based nMOF was physically modified with DSPE-PEG-FA to improve the hydrophilicity and cellular uptake by folate receptor-overexpressing cells (Choi et al., 2023). Then imiquimod (IMQ) was integrated into the MOF formulation as an immunostimulatory adjuvant, resulted in radiotherapy enhancement. In addition to MOF, hafnium based metal “X” Frameworks (MXFs) have been reported, introducing DNA “components” beyond metal ions and small organic molecules as coordination units (Yang et al., 2022). HfCl_4_ was coordinated with CpG oligodeoxynucleotides in the mixture solution of water, dimethyl sulfoxide (DMSO), and DMF to form Hf-CpG MXF, showing monodisperse and spherical nanostructures in narrow and small size distribution.

Due to the special properties of self-assembly sites between metal ions and phenolic compounds, metal phenolic networks (MPNs) exhibit specific functional moieties, redox-responsive behaviors, and reversible assembly properties (Liu P. et al., 2021). Li et al. synthesized Hb@Hf‐Ce6 NPs with a size of about 30 nm through self-assembling sites between Hf^4+^ and polyphenol (gallic acid). By coordinating with polyphenolic polymers and modification, Hf-based MPNs can reprogram tumor metabolism as well as change the tumor microenvironment, result in better cancer therapy enhancement (Li et al., 2021; Sang et al., 2021; Li et al., 2022). NCPS are generally considered to be amorphous coordination structures with reduced porosity. Liu et al. designed the photoresponsive NCP-Ce6-DOX-PEG by solvothermal method (Liu et al., 2017a). In this nanosystem, Hf^4+^ ions and presynthesized linker were dissolved in DMF and ethanol and heated at 120°C for 24 h. During this process, PVP and triethylamine (TEA) were further added as surfactant and pH regulator, respectively. The resulting NCPs exhibited a diameter of approximately 70 nm, along with porous characteristics conducive to drug (Ce6 and DOX) loading.

### 2.3 Other Hf based NMs

In addition to HfO_2_ NMs and MOFs, several investigations have synthesized Hf-based NMs through alternative methods. Recently, great attention has been paid to carbon-based NMs such as carbon dots (CDs) due to their excellent electrical conductivity, high chemical stability and low toxicity (Namdari et al., 2017). Gu, et al. embedded CDs on the surface of ZrHf-MOF to construct CDs@ZrHf-MOF nanocomposites, which exhibited strong fluorescence and rich-amino-functionalization for early diagnosis of living cancer cells (Gu et al., 2019). Notably, CDs@ZrHf-MOF was applied as scaffold for anchoring aptamer strands to recognize human epidermal growth factor receptor-2 (HER2). In another study, Su, et al. developed a one-pot pyrolysis method to prepare ultrasmall size Hf-doped CDs (HfCDs) from citric acid (CA), thiourea (TU) and HfCl_4_^54^. Orthotopic liver tumor model was established to evaluate the *in vivo* imaging performance of HfCDs, and the results showed outstanding cancer CT/FI imaging capability and rapid renal clearance. Besides carbon dots, Ma, et al. used ultrasound-assisted liquid-phase exfoliation method to successfully synthesize Hf carbide NPs with the surfactant PVP for excellent photothermal effects and anti-inflammatory properties (Ma et al., 2023).

Expanding the landscape of Hf-based NMs for cancer therapy, Chen, et al. synthesized hafnium-doped hydroxyapatite NPs (Hf:HAp NPs) by wet chemical precipitation, revealing marked γ-ray irradiation enhancement, resulting from intracellular reactive oxygen species (ROS) generation (Chen et al., 2016). Liu, et al. utilized an AIE photosensitizer for constructing a new nanoparticle system (Hf-AIE) through coordination with Hf (Liu et al., 2021c). After functionalization with DBCO, Hf-AIE-PEG-DBCO showed enhanced and prolonged accumulation at tumor site. Additionally, under X-ray irradiation, Hf-AIE-PEG-DBCO could synchronously generate effective ·OH and singlet oxygen (SO) through radiosensitization for the treatment of tumor.

## 3 Hf-based material for imaging and diagnosis

### 3.1 Hf-based material for imaging

Bioimaging provides important evidence in diagnosing cancer. However, limitations from reduced sensitivity and inadequate accumulation of contrast agents (CAs) at target sites necessitate the utilization of NM-based CAs to provide oncologists with precise anatomical information for accurate cancer staging (Siddique and Chow, 2020). As a metal element that can assist tumor imaging, Hf-NMs have been applied in various imaging modalities as functional CAs.

Iodine-based contrast agents are most used in CT imaging clinically. However, contrast based on iodine decreases with increasing tube voltage (Nowak et al., 2011). The traditional iodine-based contrast media has relatively low K-shell absorption edge (about 33 KeV), which is lower than the high X-ray tube voltages (100–140 kV) applied in CT. Therefore, high-Z elements with a higher K-edge in the range of 60–80 KeV would arouse more interest (Liu X. et al., 2017). As possible alternatives, hafnium-based contrast media have shown promising results in animal studies. In 2012, amorphous and crystalline Hf framework coated with silica were prepared for the first time, then functionalized with PEG to make the particles suitable for *in vivo* CT imaging (Dekrafft et al., 2012). Subsequent studies combined Hf-based material as a characteristic of CA with its therapeutic properties. Li, et al. designed a clearable PEGylated HfO_2_ NAs for CT imaging and enhance the radiotherapeutic effects (Li et al., 2020). Compared with the free IR780 group, the IR780/HfO_2_ NAs group showed significantly stronger fluorescence signal in 4T1 breast tumor model. At the same time, the fluorescence signal of IR780/HfO_2_ NAs group could be detected in the tumor at 24 and 48 h after injection, highest at 24 h. However, fluorescent signal of pure IR780 almost disappeared 24 h after injection. In tumor CT imaging, the CT signal of the tumor section was 47HU before injection and increased to 103HU after contrast 24 h later. Tumor sections were analyzed using TEM to determine the internalization of HfO_2_-NAs in tumor cells after intravenous administration. TEM images of tumor sections clearly demonstrate an uptake effect due to the enhanced permeability and retention (EPR) effect. This study provided a new technique of CT imaging-guided radiotherapy enhancement for further potential clinic translation. In addition, Hafnium carbon dots can also achieve rapid, tumor-targeted CT imaging in less than 1 min, providing superior developmental capabilities and longer imaging durations than iodohexol, a clinically used CT contrast agent (Su et al., 2020).

Among all available diagnostic imaging methods, X-ray remains the dominant medical imaging technology due to its wide clinical availability, high spatial resolution, rapid image acquisition, and low cost (Hsu et al., 2020). Several tissue types such as bone and lung, can be easily detected by X-ray imaging without the use of contrast agents. However, X-ray imaging is difficult to distinguish soft tissues, so contrast agents are usually required such as using oral barium suspension to achieve gastrointestinal cancer imaging. But these contrast agents have some disadvantages, especially iodinated small molecules, which limit their use to a few applications. Disadvantages include non-specific biological distribution, short blood half-life and renal insufficiency (Tepel et al., 2006). In view of these limitations, efforts have been made to develop alternative metal X-ray contrast agents, most of which are NPs with novel chemical compositions. In the field of non-NMs, studies have reported the use of Hf to synthesis X-ray contrast agent named BAY-576, an uncharged hafnium complex, experiments have confirmed that it has good X-ray imaging enhancement effect (Frenzel et al., 2016; Berger et al., 2017). McGinnity, et al. prepared controlled size HfO_2_ NPs and measured the X-ray attenuation compared with AuNPs and iodine over a range of concentrations (McGinnity et al., 2016). HfO_2_ NPs showed a better contrast than Au NPs, and they both showed significantly greater contrast efficacy than iodine. Moreover, HfO_2_ NPs exhibited stronger mid-infrared absorption, which can be used for CT contrast media and mid-infrared biosensors simultaneously. Therefore, HfO_2_ NPs could be a substitute for iodine in the future. In addition to being used as contrast agent for X-ray and CT, other studies have reported the potential of HfO_2_ NPs as mid-infrared biosensors (Ma et al., 2023). But so far, no research has been done for the application of this function in cancer imaging.

### 3.2 Hf-based material for diagnosis

In addition to cancer related imaging, Hf-NMs have significant utility in cancer diagnostics for the precise detection of biological markers within cells and bodily fluids. Presently, the majority of applications involving Hf-NMs in cancer diagnosis are focused on testing for tumor markers in saliva samples. Saliva has been reported to contain various cytokine biomarkers which associated with different cancers (Kaczor-Urbanowicz et al., 2017). Kumar, et al. first reported the fabrication of HfO_2_ based immunosensor based on anti-CYFRA-21-1 for efficient detection of CYFRA-21-1 in saliva samples (Kumar et al., 2016). Later, based on this work and application of nanostructured metal oxide (NMO) based cancer biosensor, this team used electroactive reduced graphene oxide (RGO) modification to inhibit Hf NPs agglomeration to design an efficient and label free immunosensor based on HfO_2_@RGO. The fabricated immunoelectrode have wider linear detection range, higher sensitivity, and remarkable lower detection limit of CYFRA-21-1 in oral cancer patients (Kumar et al., 2018). This work contributes for the development of capturing the Brownian motion of nonagglomerated NPs and contributes to highly efficient biosensing platforms for the detection of cancer, infectious diseases and pathogens. Hao, et al. explored a graphene-based integrated portable nanosensing system based on HfO_2_ NPs, the size of which is smaller than a phone. This graphene-based field effect transistor (GFET) employed a golden electrode on the substrate, buried by a 30 nm HfO_2_ NPs dielectric layer serving as the gate, thus eliminating the need for the external gate electrode. In addition, because of the high permittivity of HfO_2_ NPs (*ε* = 16), insecurely high operating gate voltages was needless. Interleukin-6 (IL-6) was used as a representative marker to examine the sensing capability (Hao et al., 2019). The nanosensing system responds to the change of IL-6 concentration in saliva within 400s with a detection limit down to 12 p.m. This study provided a potential use for daily cytokine biomarkers detection for early cancer diagnosis. In the future, research can explore a wider array of tumor markers, encompassing, but not limited to, cytokines and proteins, as targets for detection in diverse bodily fluid samples (such as serum, urine, plasma, cerebrospinal fluid, *etc.*) in different clinical scenarios.

## 4 Hf-based material for cancer therapy

The era of disease theranostics involving Hf-based metal NPs has emerged. Hafnium, as a high-Z element, has spurred researchers to explore its application in the field of cancer. These NPs not only hold promise for tumor visualization and diagnosis but also for cancer therapy, capitalizing on their unique structures, functionalities, and chemical properties. Currently, Hafnium-based cancer monotherapy predominantly focuses on enhancing radiotherapy and X-ray-induced PDT. Meanwhile, combined therapy involves amalgamating radiotherapy with additional treatments such as thermal therapy, chemotherapy and immunotherapy.

### 4.1 Hf-based material for radiotherapy

Radiotherapy has become a standard treatment for cancer, yet it faces limitations such as low radiation absorption and drug resistance. Due to hafnium’s high atomic weight, electron density, and chemical stability, Hf-NMs exhibit the potential to function as radiotherapy sensitizers. NBTXR3, the first commercialized nanomaterial, was developed for direct local intratumoral (i.t.) injection and subsequent radiosensitization. In different tumor models, NBTXR3 showed a clear advantage over radiotherapy alone in terms of survival, tumor-specific growth delay, and local control (Maggiorella et al., 2012). NBTXR3 has been approved in European marketing for radiotherapy to soft tissue sarcoma.

Except NBTXR3, there are also some preclinical Hf NPs used for radiotherapy. The HfO_2_ NAs designed by Li et al. can kill cancer cells by generating free-radical generation upon X-ray irradiation. In addition, the PEGylated HfO_2_ NAs exhibited effective tumor homing ability by intravenous injection and demonstrated by HfO_2_ NA-enhanced CT imaging in a 4T1 breast tumor model. Radiotherapy with HfO_2_ NAs treatment induced the most significant cancer cell apoptosis and necrosis, whereas radiotherapy alone produced only moderate damage to tumor cells (Li et al., 2020). This study indicates that Hf-based NMs may simultaneously enhance imaging and therapy, thereby integrating cancer diagnosis and treatment.

**FIGURE 1:**
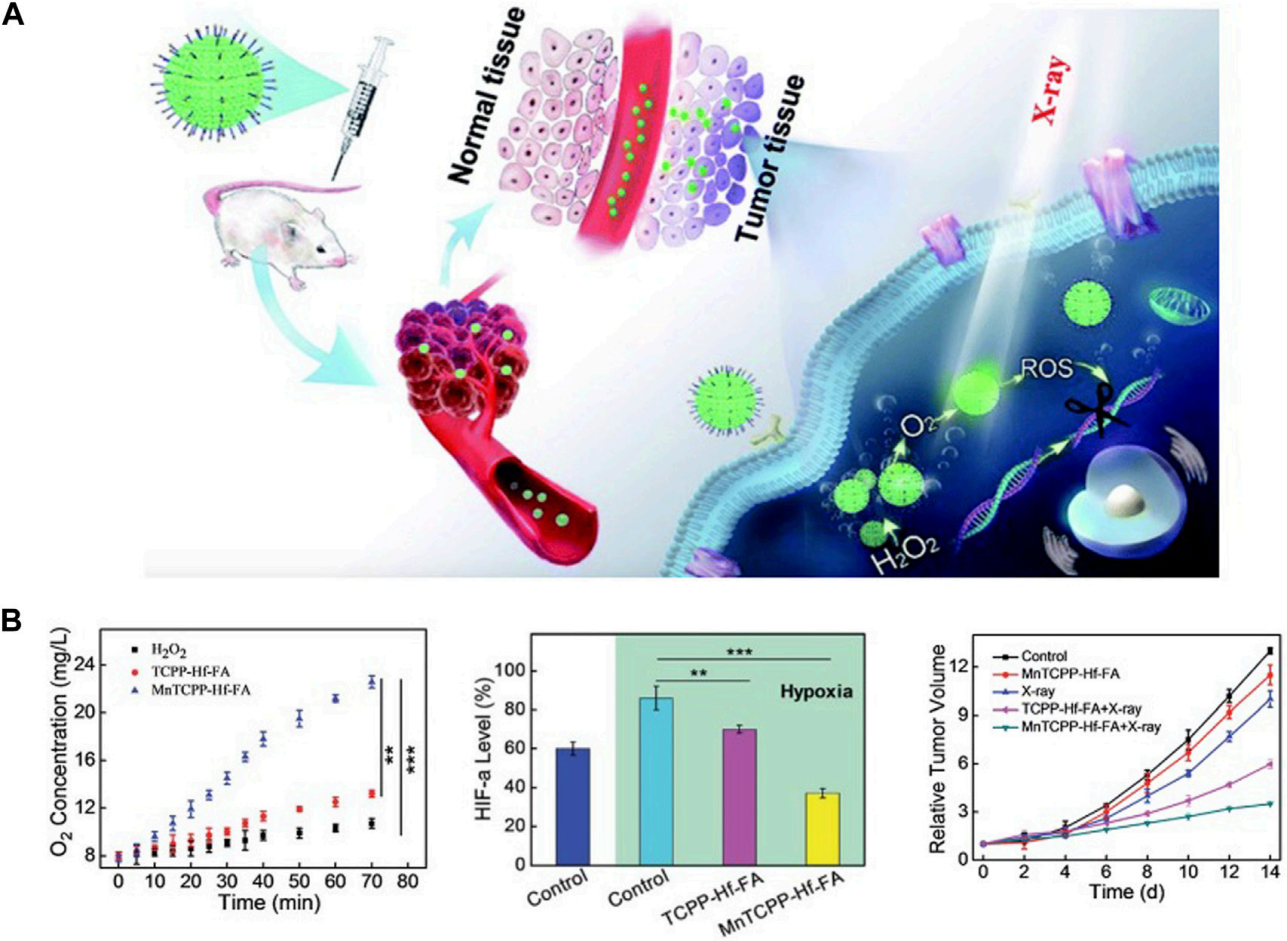
Hf-based material for radiotherapy. **(A,B)** MnTCPP–Hf–FA MOF NPs can overcome hypoxia-induced radioresistance and prevent postoperative recurrence. Copyright 2019, The Royal Society of Chemistry.

Several hafnium-based MOFs/NCPs have also been designed for radiotherapy enhancement. To improve the effectiveness of radiotherapy against hypoxic tumors, MnTCPP-HF-FA MOF NPs were designed as a catalase like nanosensitizer to decompose endogenous H_2_O_2_ to O_2_, inhibit HIF-1α activation to enhance radiotherapy (Chen et al., 2019). *In vivo*, this MOF NP could effectively inhibit melanoma growth and prevent tumor recurrence after only radiotherapy once by intravenous administration (Figure 1). In another study, UiO-66-NH_2_ (Hf) also induced cell apoptosis by inducing DNA damage and increasing ROS levels (Zhou et al., 2022), A single injection of UiO-66-NH_2_ (Hf) in combination with X-ray irradiation significantly inhibited tumor growth in a xenograft model. This single local treatment procedure avoids exposure of normal tissues to X-rays as much as possible and minimizes side effects of ionizing radiation, which is very important in clinical translation. Due to the porous property of MOFs, it is easy to realize the loading of functional components such as drugs and photosensitizers, so this may be a direction for the design of combination therapy.

Up to now, a large number of literatures have reported hafnium-based material enhanced radiotherapy. Generally, these materials significantly augment treatment efficacy, achieving enhanced tumor eradication by generating ROS and disrupting cell DNA. Beyond radiotherapy, there exists a multitude of treatment modalities for tumors. As foundational and translational research progresses, innovative material designs that cater to diverse treatment approaches could serve as a guiding force for future developments.

### 4.2 Hf-based material for PDT

PDT represents a novel tumor treatment modality characterized by its high safety profile and minimal side effects. It operates by activating photosensitizers to induce ROS-mediated destruction of tumor cells (Sun W. et al., 2020; Chen et al., 2020). However, most PDT penetration depth is less than 2 cm, which cannot meet the clinical scenario. This limitation can be reached by using high penetrating X-ray as the excitation source. The advantages of X-ray induced PDT (X-PDT) over other PDT lie in the clinical potential of treating deep-seated cancers, such as carcinoma *in situ*, and the improvement of treatment efficiency under lower dose of X-ray compared with radiotherapy (Kamkaew et al., 2016; He et al., 2022). In the research of X-PDT photosensitizers, inorganic NMs like high-Z metals have become ideal materials for nanoscintillators because they can effectively absorb the high energy of radiated photons (Fränzel and Gerlach, 2009; Stanton et al., 2014). Till now, high-Z metal NMs such as hafnium NPs have been studied to perform great X-PDT effectiveness (Zhang et al., 2023).

**FIGURE 2:**
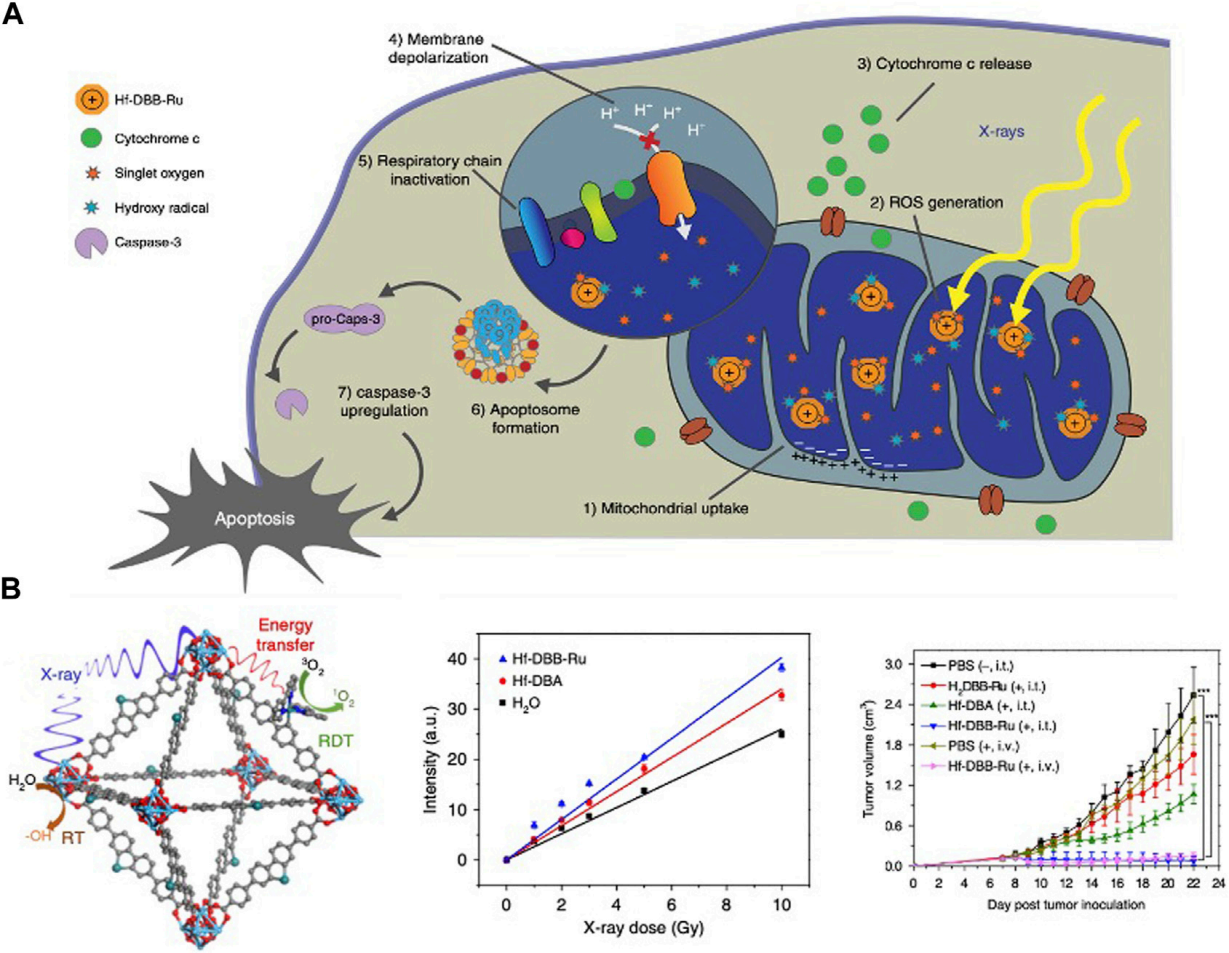
Hf-based material for photodynamic therapy. **(A,B)** Hf-DBB-Ru targeted mitochondria, generated • OH and singlet oxygen to treat deep tumors. Copyright 2018, Nature Portfolio.

In 2014, Lin et al. reported the research on the application of hafnium in X-ray absorption (Wang et al., 2014). Later, the team synthesized Hf-DBB-Ru with mitochondrial targeting ability. By X-PDT, Hf-DBB-Ru generated • OH from Hf6 secondary building units (SBUs) and singlet oxygen from DBB-Ru photosensitizer, depolarized mitochondrial membranes and initiate apoptosis of tumor cells (Ni et al., 2018) (Figure 2). In 2018, the same team synthesized nMOF DBP-Hf and TBP-Hf, the X-ray energy absorbed by the Hf SBUs is directly transferred to the anthracene ligand by the inelastic scattering of photoelectrons. Combined with IDOi to improve the comprehensive treatment effect, nMOF achieved 100% cure of breast and colorectal cancer (Lu et al., 2018).

By reducing the dimensional formation of nMOF, this group integrated iridium and ruthenium based photosensitizer (Hf-BPY-Ir or Hf-BPY-Ru) with high singlet oxygen yield into the hafnium based two-dimensional nanoscale Metal Organic Layer (nMOL) (Lan et al., 2017), X-PDT has been achieved to treat deep tumors. In experiments of simulating deep tumor, the PDT control group stimulated by visible light cannot show anti-cancer activity because the energy cannot reach the photosensitizer, while the X-PDT group still maintained significant efficacy. One year later, two new structures, Hf12-Ir and Hf6-Ir were reported. At low doses of X-ray irradiation, Hf12 or Hf6 SBU was shown to efficiently generate hydroxyl radicals, which the tumor regression rate reached 99% by X-PDT (Lan et al., 2018).

Unlike radiotherapy, PDT has limited clinical applications, particularly when it comes to NMs based on high-Z metal elements. Most research remains confined to the laboratory, albeit substantial results have been showcased in animal models.

### 4.3 Hf-based material for combined therapy

In numerous cases, the antitumor outcomes of combination therapy do not simply entail an additive effect, where the results of each therapy are merely summed. Instead, they frequently manifest a synergistic effect (Carvalho et al., 2021). Hf-based NPs have been successfully used in cancer radiotherapy. Furthermore, the multiple physical, chemical, and catalytic properties enable Hf-based NPs to achieve satisfactory therapeutic effects for cancer combination therapy.

#### 4.3.1 PTT-radiotherapy

**FIGURE 3:**
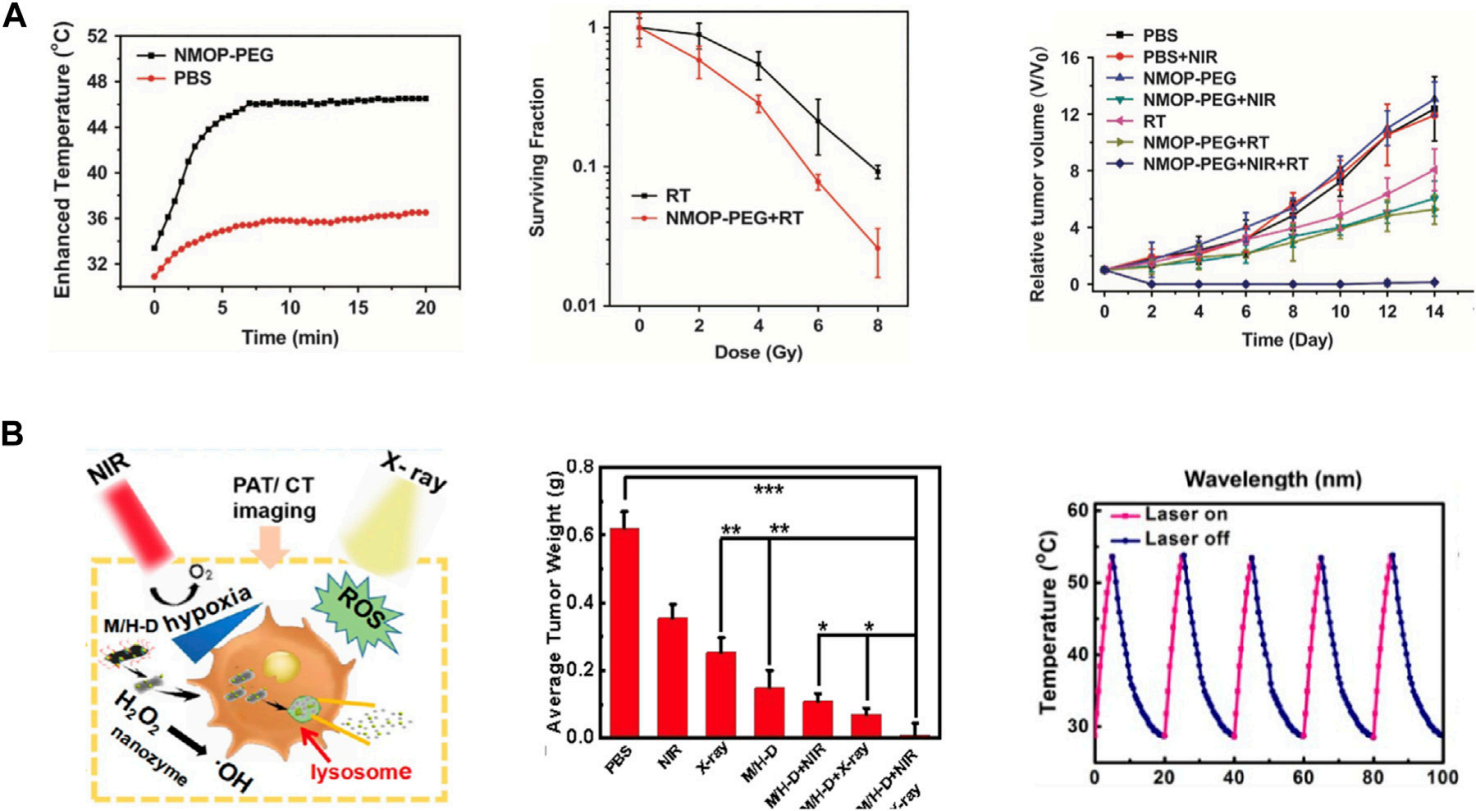
Hf-based material for Thermal Therapy-Radiotherapy. **(A)** Mn/Hf-IR825@PDA-PEG for multimodal imaging and synergistic thermo-radiotherapy. Copyright 2017, Nature publishing group. **(B)** TME-responsive M/H-D achieve precise nanoradiosensitization, potent oxygenation in tumor and efficient suppression to tumor. Copyright 2020, American Chemical Society.

PTT-radiotherapy refers to the combination of PTT and radiotherapy for cancer treatment (Yang et al., 2016). The high temperature produced by PTT can destroy cancer cells and improve the effect of radiotherapy (Song et al., 2016). In monotherapy, hafnium carbide NPs have been designed to achieve PTT with noninflammatory cancer treatment (Ma et al., 2023). Moreover, thermal effect can induce DNA double strand break and inhibit the repair of DNA damage caused by radiation. Besides, PTT promotes blood circulation in the tumor microenvironment and alleviates tumor hypoxia, thus reduces radiotherapy resistance of cancer cells. In addition, hyperthermia mainly kills tumor cells at S stage, while tumor cells at G2/M stage are more sensitive to radiation (Mao et al., 2016). Therefore, the combination of PTT and radiotherapy is of great significance in cancer treatment. Yang et al. synthesized core-shell and co-doped Mn/Hf-IR825 NMOP prepared by a post-synthetic cation exchange method for the combination of PTT and radiotherapy (Yang et al., 2017b). Mn^2+^ ions are used as contrast agents for T1-weighted MR Imaging; IR825 was used as an excellent contrast agent for photoacoustic (PA) imaging as well as a photothermal agent for PTT; Hf^4+^ provides contrast for X-ray computed tomography (CT) imaging and enhance the efficacy of radiotherapy. Mn/Hf-IR825 NMOP achieve multimodal image, treated tumors synergistically by PTT and radiotherapy and had no long-term toxicity in treated mice (Figure 3A). Bao, et al. designed another bimetal Hf/Mn MOF nanoparticle, the MRI imaging characteristic of Mn and CT enhancer characteristic of Hf are utilized to achieve CT/MRI dual-mode imaging, which can better monitor cancer treatment (Bao et al., 2020). Under irradiation with 808 nm lasers, dramatic temperature increases were recorded for the NPs solution and cancer region of i.v. injected animal model. In this work, targeting MRI/CT also contrast enhanced imaging and imaging-guiding PTT-radiotherapy synergistic therapy. Fu, et al. developed an efficient nanoradiosensitization system that enhances the radiation doses in cancer cells to sensitize radiotherapy with PTT (Fu et al., 2020). MoS_2_/HfO_2_-Dextran (M/H-D) was used to degrade and release the HfO_2_ NPs in tumor microenvironment and enhance tumor penetration of the HfO_2_ NPs. Then, PTT increased peroxidase-like catalytic efficiency of the M/H-D nanoradiosensitizer in tumor microenvironment, which selectively catalyzed intratumorally overexpressed H_2_O_2_ into highly oxidized hydroxyl radicals (·OH), to overcome hypoxia-associated radiotherapy resistance. The heat induced by PTT also relieved the intratumoral hypoxia to sensitize radiotherapy (Figure 3B).

The above three works successfully utilized Hf-based NPs for synergistic PTT-radiotherapy. PTT has been reported to have multiple functions, all these changes contribute to the high sensitivity of tumor cells to radiotherapy, thereby reducing damage to surrounding normal tissue (Xu and Pu, 2021).

#### 4.3.2 Chemotherapy-radiotherapy

**FIGURE 4:**
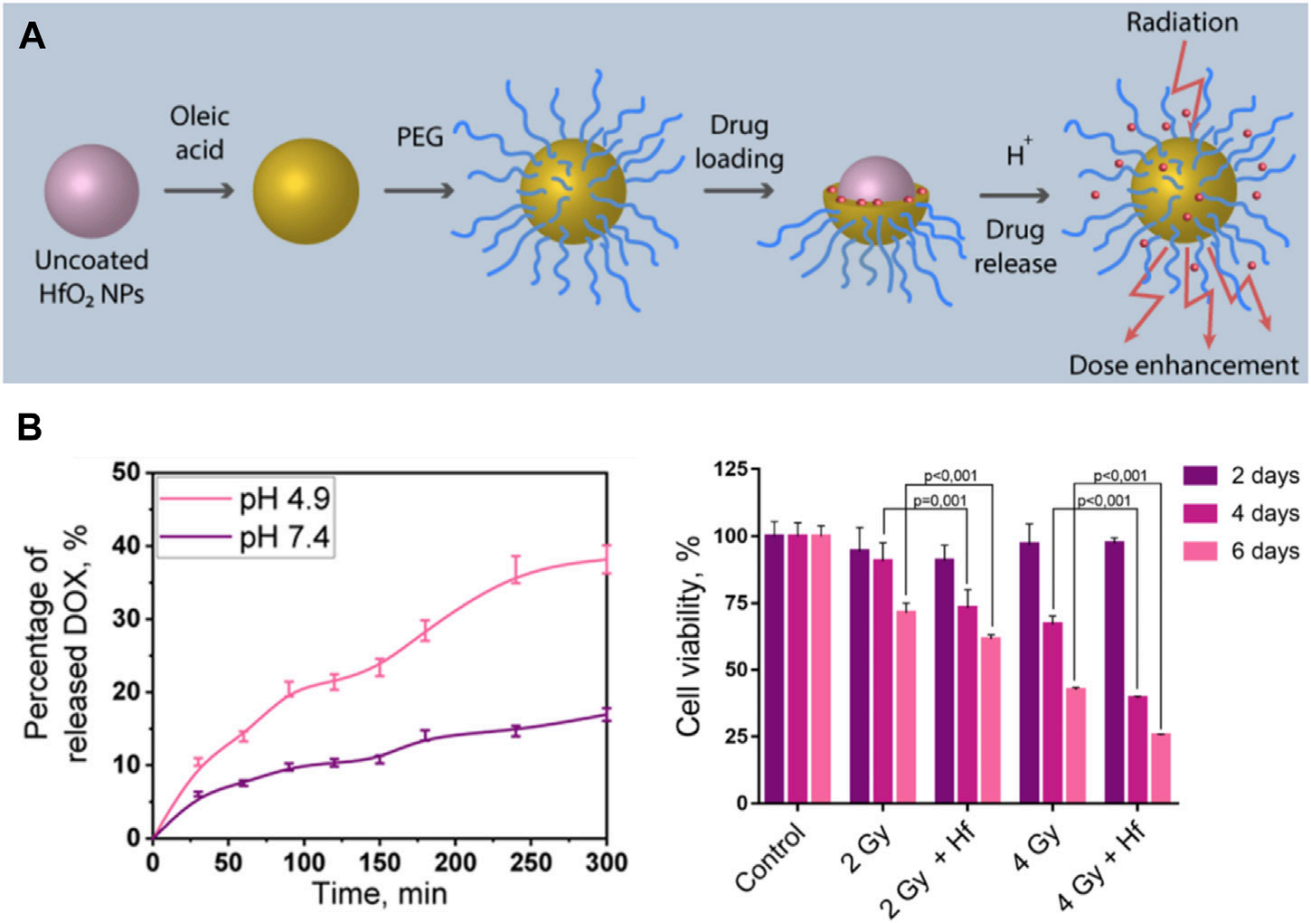
Hf-based material for chemotherapy-radiotherapy. **(A,B)** Doxorubicin (DOX) loaded HfO_2_ nanoparticles for enhanced chemoradiotherapy. Copyright 2021, American Chemical Society.

Radiotherapy and chemotherapy have become the two most commonly used cancer treatments clinically besides surgery (Siegel et al., 2022). Chemo-radiotherapy, which combines chemotherapy with radiotherapy, has been applied clinically since the 1970s and have shown synergistic effects (Nagasawa et al., 2021). Some chemotherapy drugs have radiosensitization function, in addition, radiotherapy can inhibit local tumor growth, while chemotherapy can control systemic metastatic tumors. Therefore, it is feasible to combine chemotherapy with multifunctional radiosensitizer based on high Z NMs to achieve the synergistic effect of chemotherapy and radiotherapy to cure tumor. Liu et al. fabricated a pH-responsive Hf-based NCP, loaded with the chemotherapeutic drug TPPGC (Liu et al., 2017b). They found that the prepared NPs could depolymerize and release TPPGC under an acidic tumor microenvironment, which can arrest the cancer cell cycle into the radiation sensitive phase (G1). Sherstiuk, et al. designed Hafnium Oxide-based NPs with DOX loaded in, evaluated the efficacy of the nanoplatform as an agent for the combined chemoradiotherapy (Sherstiuk et al., 2021). The DOX loaded nanoplatform had a lower IC50 than pure DOX, demonstrate the NPs accumulate more effectively inside the cells than DOX alone (Figure 4). However, its anti-tumor effect *in vivo* has not been further investigated. In another study, a combination of poly (ADP-ribose) polymerase inhibitor (PARPi) talazoparib and chemotherapeutic temozolomide drug-loaded Hf-BDC nMOF was designed to investigate the therapeutic effect (DuRoss et al., 2021). These P-selectin targeted and fucoidan surface functionalized nMOFs showed improved tumoral accumulation and subsequently enhanced therapeutic effects in a heterotopic, syngeneic colorectal cancer model. More importantly, loading chemotherapeutic drugs on nMOF modified by targeted agents can reduce unnecessary drug toxicity, which showed better clinical application prospects.

Aiming at therapy enhancement of NBTXR3, Nanobiotix used various cancer cell lines and tried different drug combinations. Results showed that cisplatin-based chemotherapy combined with NBTXR3 based radiotherapy significantly improved cancer cell destruction (Zhang et al., 2021). These existing research results indicate that the combination of industrialized radiotherapy sensitizer such as NBTXR3 with clinically used chemotherapeutic drugs can produce better synergistic treatment. In the laboratory, loading Hf-NMs with chemotherapeutic drugs may be helpful in tumor targeting due to the properties of Hf and decoration of NMs. It is believed that there will be more studies on combining Hf-enhanced radiotherapy with chemotherapy soon.

#### 4.3.3 Immunotherapy-radiotherapy

Radiotherapy and immunotherapy are effective treatment for cancers. However, most patients do not respond to currently available immunotherapies based on immune checkpoint inhibitors (ICIs) (Hegde and Chen, 2020). Benefit of immunotherapy is limited to patients who have a pre-existing active immune tumor microenvironment that can be reactivated by ICIs (Turgeon et al., 2019). It is now recognized that radiotherapy kills cancer cells as well as changes the tumor microenvironment, enhancing cell recognition by the immune system (Wang and Haffty, 2018). Radiotherapy increases the expression of tumor-associated antigen, stimulates recruitment of dendritic cells and activate cytotoxic CD8^+^ T cells (Gupta et al., 2012). This immunological cascade generates T cells to induce immunogenic cell death directed against cancer cells (Zhu et al., 2021). By this ability, radiotherapy provides a cost-effective approach to potentially improve the response to immunotherapy. In 2016, a study presented a list of 93 clinical trials combining radiotherapy with CTLA-4, PD-1, PD-L1, or other immune molecule drugs (Kang et al., 2016). *ClinicalTrials.gov* now reports more than 200 completed or recruiting trials evaluating new combined therapy. In the field of nanomedicine, radiotherapy combined/enhanced with immunotherapy is also becoming a hot topic.

**FIGURE 5:**
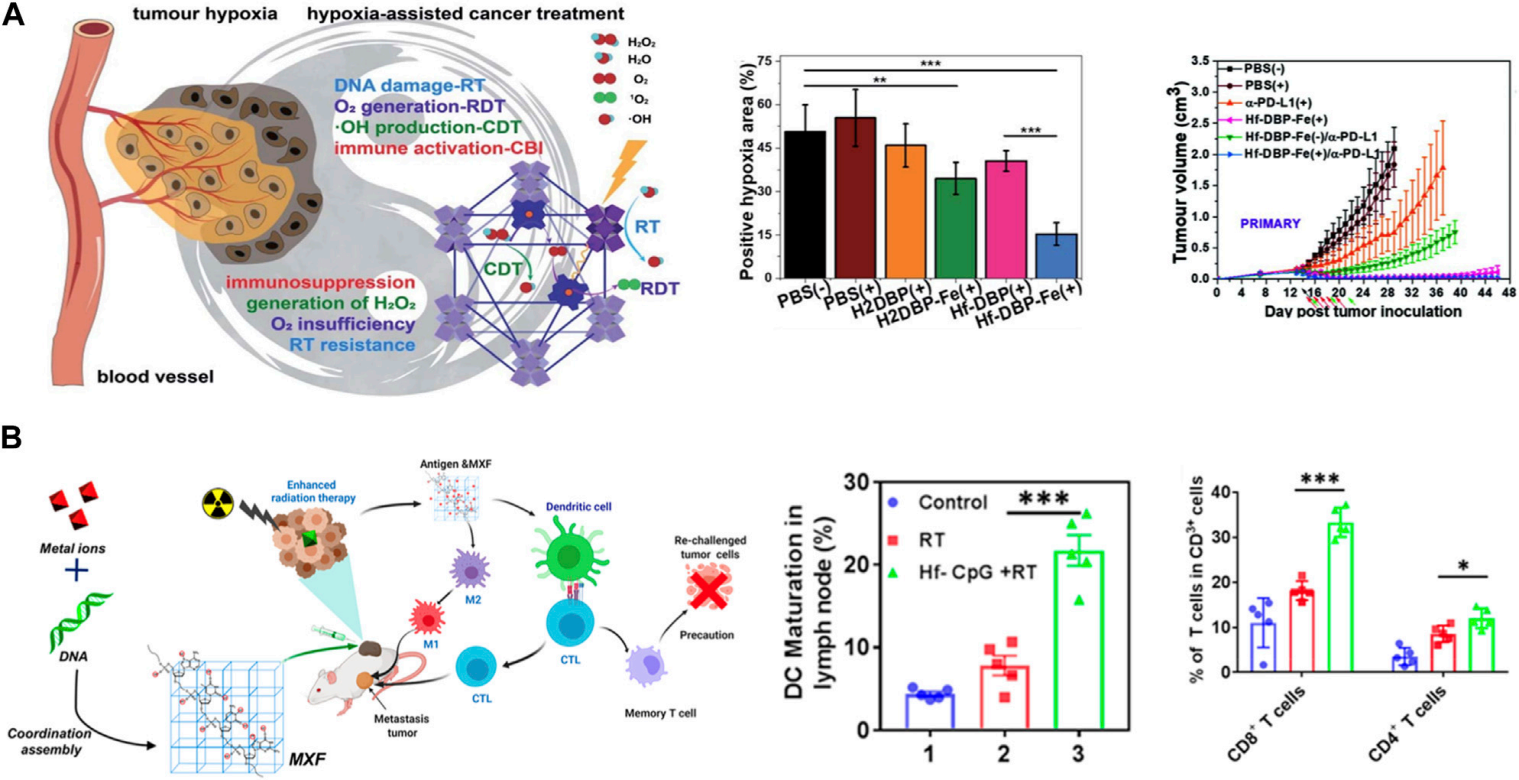
Hf-based material for immuno-Radiotherapy. **(A)** nMOF Hf-DBP-Fe enhanced radiotherapy with low-dose X-ray irradiation, leading to highly immunogenic tumor microenvironments for synergistic therapy. Copyright 2020. The Royal Society of Chemistry. **(B)** Hf-CpG MXF achieved enhanced radiotherapy and further trigger robust tumor-specific immune responses. Copyright 2022, American Chemical Society.

Ni et al. fabricated a biomimetic MOF HF-DPP-Fe with catalase-like activity for immunoradiotherapy (Ni et al., 2020) (Figure 5A). HF-DPP-Fe MOF can catalyze H_2_O_2_ to generate oxygen and hydroxyl radicals, thereby improving the hypoxic area of tumor microenvironment, promoting the effect of radiotherapy. The induced hyperimmunogenic tumor microenvironment offers potential for tumor immunotherapy, and the synergistic effect of anti-PD-L1 immune checkpoint blockade not only eliminates primary tumors, but also inhibits the growth of distant tumors. There are also other works using HF-based MOF achieved immunoradiotherapy through different modifications, such as the oxygen delivering by hemoglobin and reprogramming oxygen metabolism in tumor (Sang et al., 2021; Li et al., 2022). In addition, studies have found that radiotherapy can increase the infiltration of T cells, thereby improving the efficacy of immunotherapy. Some small molecular inhibitors of immune checkpoints can be loaded into porous Hf-based MOF structures and realize immunoradiotherapy combination. Lu, et al. loaded IDOi into a Hf-based MOF (IDOi@DBP-Hf), achieved effective growth inhibition and even eradication of local and distal tumors under low-dose irradiation (Lu et al., 2018). The release of IDOi effectively increased the infiltration of functional CD8^+^ T cells in tumors, overcoming some of the limitations of checkpoint blockade immunotherapy, also demonstrated that Hf-NMs can be used for radiotherapy synergized with immunotherapy and achieve the purpose of simultaneous treatment of primary and metastatic lesions. Apart from combining treatment with immunotherapy, there are also studies focusing on the activation of autologous anti-tumor immunity by radiotherapy. Yang et al. designed a hafnium-based metal “X” frameworks Hf-CpG to induce ICD by enhancing radiotherapy to treat the primary tumor, which promotes innate anti-tumor immunity, such as increasing CD8^+^ T cells, dendritic cell maturation and M1 polarization of macrophages, achieves the treatment of tumor metastases, and produces long-term anti-tumor immune memory (Yang et al., 2022) (Figure 5B).

In a recent study, Liu, et al. prepared Hf-nIm@PEG by the coordination of hafnium ions with 2-nitroimidazole (2-nIm), and then modified with PEG (Liu N. et al., 2023). Under X-ray irradiation, NO can be released from 2-nIm, which can relieve the hypoxic immunosuppressive TME to sensitize radiotherapy. Additionally, NO can also react with superoxide ions to generate reactive nitrogen species (RNS) to induce cell apoptosis. More importantly, researchers discovered that Hf^4+^ can effectively activate the cyclic-di-GMP-AMP synthase (cGAS)-stimulator of interferon genes (STING) pathway to achieve enhanced radio-immunotherapy of inhibiting cancer progression, metastasis, and recurrence. Also, aiming at blocking radiation DNA repair and activating cGAS-STING pathway, Liu, et al. designed HfO_2_ NPs coated Ataxia-telangiectasia mutated and RAD3-related (ATR) kinase inhibitor (ATRi), can efficiently trigger systemic antitumor immune response via driving immune cell infiltration and enhancing immunogenicity based on the cGAS-STING pathway (Liu R. et al., 2023).

#### 4.3.4 Sonodynamic-radiotherapy

Sonodynamic therapy (SDT) is an effective treatment technique for deep cancer (Yang et al., 2019). During SDT, ultrasound can irradiate sonosensitizers to generate reactive oxygen species to kill cancer cells (Son et al., 2020). Therefore, it can be expected that the combination of SDT and RT may simultaneously improve the efficacy of low-dose RT and improve the penetration depth of SDT.

Wang et al. reported Hf-hemoporfin frameworks (HfHFs) for synergetic sonodynamic and radiotherapy of deep cancer (Wang et al., 2022). Under ultrasound, HfHFs can be triggered to produce singlet oxygen (^1^O2) due to the sonodynamic effect. Under X-ray irradiation, Hf4^+^ in HfHFs can absorb X-rays and generate hydroxyl radicals (OH) through radiotherapy. Thus, the HfHFs nanoplatform exhibits higher ROS production capacity with mono-sonosensitizers and mono-radiosensitizers. In addition, HfHFs can be used as novel CT contrast for cancer imaging. So far, there are still few studies focus on SDT-RT. More combined therapies should be developed based on the advantages of SDT.

## 5 Translation, toxicity and safety for Hf-NMs

Searched through *clinicaltrials.gov*, by 2022, clinical trials (15 records) have been performed using NBTXR3 for treatment in combination with radiotherapy, most of the major locations are located in the United States and France (Table 2). The earliest phase 1 trial of NBTXR3 (NCT01433068) started in 2011, results showed that i.t. injection of NPs (22 patients with sarcoma in France) combined with external beam radiation therapy resulted in a significant reduction in tumor volume. The patients all well tolerated, and the NPs did not leak into adjacent healthy tissue (Bonvalot et al., 2017). Besides, NBTXR3 have since been applied in a variety of clinical scenarios, including neoadjuvant therapy of tumors, to reduce tumor size for 6–8 weeks combined with radiotherapy before surgery (Pottier et al., 2015; Zheng et al., 2021). In 2019, a prospective phase II-III study (NCT02379845) was published on Lancet Oncology, compared the efficacy and safety of NBTXR3 combined with radiotherapy and radiotherapy alone in neoadjuvant treatment of locally advanced soft tissue sarcoma (Bonvalot et al., 2019). 180 patients were randomly assigned to receive NBTXR3 plus radiotherapy (n = 90) or radiotherapy alone (n = 90), the results showed that NBTXR3 plus radiotherapy group achieved more complete pathological response (residual tumor <5%), while adverse events were similar in two groups. To date, NBTXR3 has started been further explored in different kinds of solid tumors, including head and neck cancer, rectal cancer, hepatocellular carcinoma (liver cancer), prostate cancer, and soft tissue sarcoma.

**TABLE 2:** Clinical trials of NXTBR3.

Row	Study title	Status	Conditions	Interventions	Major location
1	NBTXR3 Activated by Radiation Therapy for the Treatment of Locally Advanced or Borderline-Resectable Pancreatic Cancer	Recruiting	Pancreatic cancer	Other: NBTXR3	M D Anderson Cancer Center, United States
				Radiation: Radiation Therapy	
2	NBTXR3 and Radiation Therapy for the Treatment of Inoperable Recurrent Non-small Cell Lung Cancer	Recruiting	Lung Cancer	Other: NBTXR3	M D Anderson Cancer Center, United States
				Radiation: Radiation Therapy	
3	NBTXR3 Crystalline Nanoparticles and Stereotactic Body Radiation Therapy in the Treatment of Liver Cancers	Terminated	Liver Cancer	Radiation: NBTXR3, IL or IA injection + SBRT	CHU La Croix Rousse, France
4	NBTXR3 Crystalline Nanoparticles and Radiation Therapy in Treating Patients With Soft Tissue Sarcoma of the Extremity	Completed	Adult Soft Tissue Sarcoma	Device: NBTXR3	Institut Bergonie, France
5	NBTXR3 Nanoparticles and EBRT or EBRT With Brachytherapy in the Treatment of Prostate Adenocarcinoma	Terminated	Prostate Cancer	Drug: NBTXR3 activated by IMRT only	Dana Farber Cancer Institute/Brigham and Women’s Hospital, United States
				Drug: NBTXR3 activated by Brachytherapy & IMRT	
6	NBTXR3, Radiation Therapy, and Pembrolizumab for the Treatment of Recurrent or Metastatic Head and Neck Squamous Cell Cancer	Recruiting	Head and Neck Squamous Cell Carcinoma	Other: NBTXR3	M D Anderson Cancer Center, United States
				Radiation: Hypofractionated Radiation Therapy	
				Biological: Pembrolizumab	
				Radiation: Stereotactic Body Radiation Therapy	
7	NBTXR3, Chemotherapy, and Radiation Therapy for the Treatment of Esophageal Cancer	Recruiting	Esophagus Adenocarcinoma	Drug: Capecitabine	M D Anderson Cancer Center, United States
				Drug: Carboplatin	
				Drug: Docetaxel	
8	NBTXR3 Activated by Radiotherapy for Patients With Advanced Cancers Treated With An Anti-PD-1 Therapy	Recruiting	Microsatellite Instability-High Solid Malignant Tumour	Drug: NBTXR3	Christiana Care Health Services, United States
				Radiation: SABR	
				Drug: Nivolumab	
				Drug: Pembrolizumab	
9	NBTXR3 Crystalline Nanoparticles and Radiation Therapy in Treating Randomized Patients in Two Arms With Soft Tissue Sarcoma of the Extremity and Trunk Wall	Completed	Soft Tissue Sarcoma	Device: NBTXR3	Capital Region Cancer Service, Canberra Hospital Canberra, Australia
				Device: Radiation therapy	
10	NBTXR3 and Radiation Therapy in Treating Patients With Locally Advanced SCC of the Oral Cavity or Oropharynx	Recruiting	Head and Neck Cancer	Device: NBTXR3 activated by IMRT	Centre Francois Baclesse, France
11	NBTXR3, Radiation Therapy, Ipilimumab, and Nivolumab for the Treatment of Lung and/or Liver Metastases From Solid Malignancy	Not yet recruiting	Malignant Solid Neoplasm	Other: NBTXR3	M D Anderson Cancer Center, United States
				Biological: Ipilimumab	
				Biological: Nivolumab	
				Radiation: Radiation Therapy	
12	Re-irradiation With NBTXR3 in Combination With Pembrolizumab for the Treatment of Inoperable Locoregional Recurrent Head and Neck Squamous Cell Cancer	Active, not recruiting	Head and Neck Squamous Cell Carcinoma	Other: NBTXR3	M D Anderson Cancer Center, United States
				Procedure: Intensity-Modulated Proton Therapy	
				Radiation: Intensity-Modulated Radiation Therapy	
13	NBTXR3 With or Without Cetuximab in LA-HNSCC	Recruiting	Head and Neck Squamous Cell Carcinoma	Drug: NBTXR3	Lee Moffitt Cancer Center and Research Institute Tampa, United States
				Drug: Cetuximab	
				Radiation: Radiation Therapy	
14	A Study of PEP503 With Radiotherapy in Combination With Concurrent Chemotherapy for Patients With Head and Neck Cancer	Terminated	Head and Neck Squamous Cell Carcinoma	Drug: PEP503	Keelung Chang Gung Memorial Hospital, Taiwan
				Drug: Cisplatin	
				Radiation: Radiotherapy	
15	A Study of PEP503(Radio-enhancer) With Radiotherapy and Chemotherapy for Patients With Rectal Cancer	Terminated	Rectal Cancer	Drug: PEP503	Kaohsiung Medical University Hospital, Taiwan
				Drug: 5-fluorouracil	
				Drug: capecitabine	

Previous preclinical studies on radiotherapy enhancement of Hf-based NPs mainly relied on i.t. injection (Bonvalot et al., 2017; Bonvalot et al., 2019), the i.v. injection of reported Hf-based NPs has not been carefully studied so far. I.t. uptake of NPs performs more efficient (Rancoule et al., 2016), however, considering clinical transition, not all solid tumors are available to be inject NPs i.t. because of deep location, better delivery routes and tumor-targeting approaches are needed (Yang et al., 2014). In addition to endoscopic treatment for gastrointestinal tumors, A doctor in the United States has treated a patient with pancreatic ductal adenocarcinoma by EUS-guided intratumoral injection of NBTXR3 (Bagley et al., 2022). The patient received NBTXR3 by local endoscopic delivery without any acute adverse events and was subsequently treated with radiotherapy. This attempt demonstrates initial feasibility of local endoscopic delivery of NBTXR3 and other NMs. In addition to gastrointestinal tumors, many intraluminal solid tumors can be administered in a similar manner, such as esophageal, ureteral/bladder tumors, etc (Xiao et al., 2019; Shakeri-Zadeh et al., 2021).

Intravenous agents remain better choices for tumors in hard-to-reach sites and for systemic/metastatic tumors (Yong et al., 2019). It requires higher safety of materials, and the designed drugs need better tumor homing ability. In the future, advanced tumor-targeted Hf-based NMs for i.v. injection should be obtained by means of aptamers and other methods, to help these patients receive enhanced radiotherapy and combined therapy.

*In vitro* and vivo studies, cytotoxicity depends on the nanoparticle size, concentration, and administration. In general, NPs are thought to cause (in the absence of irradiation): production of reactive oxygen species, DNA damage, disruption of mitochondrial activity and cell cycle (Mahmoudi et al., 2011; Arora et al., 2012). Another important factor is the elimination of these NPs, depending on their size and the body’s ability to eliminate. NMs smaller than 10 nm can be removed by renal filtration. Without degradation, large NMs will accumulate in the body, especially in the reticuloendothelial system, causing late side effects with potential residual toxicity. The main organs of NPs accumulation are the liver, bones, spleen, blood, lymphatic system, and thyroid. Several studies have previously investigated the cytotoxicity and safety of bare and surface functionalized HfO_2_ NPs *in vitro*. HfO_2_ NPs were reported to be nontoxic to a variety of cell types at concentrations <2 mg/mL^13^. McGinnity, et al. reported 60–90 nm HfO_2_ NPs to have colloidal stability in DI water for 2 days, and in cell culture media for at least 10 days without agglomerating or settling (McGinnity et al., 2021), showing good biological stability. Among the Hf-NMs mentioned previously, the diameter of most synthetic NPs can reach 100 nm or smaller, with low toxicity *in vitro* cell CCK-8 experiments. *In vivo* animal experiments, the stability of many NPs reached the standard of intravenous injection, especially the MOF-based NMs. In addition, the toxicity of NPs accumulation in different organs was evaluated in studies, satisfactory results were achieved.

Owing to the ultimate purpose of Hf-based NMs is clinical translation, like NBTXR3, it is particularly important to evaluate their biosafety and complications among patients. In reported clinical trials, the most common adverse event related to NBTXR3 administration was pain, followed with low blood pressure, feeling hot, and oedema peripheral (Bonvalot et al., 2017; Bonvalot et al., 2019). In short, the patient’s side effects were sporadic and manageable. In order to broaden NBTXR3 to more clinical scenarios, Casal et al. injected NBTXR3 into mediastinal and hilar lymph nodes (LN) in pig model, the NPs were retained in 100% of LN at 30 min and 90% of LN at 8 days (Casal et al., 2020). Ultrasound-guided injection achieved a high rate of nanoparticle retention, low extravasation, and no visible nanoparticle embolization. The study establishes a foundation in a large animal model as a potential precursor to clinical trials.

## 6 Conclusion and prospective

In this review, we summarize the application of hafnium-based NMs in cancer. The rapid development of nanotechnology makes it possible to diagnose and treat cancer based on NMs. Hf-based NPs have good biosafety and can be used as contrast agent in cancer imaging and radiotherapy sensitization. Combining radiotherapy with other cancer therapy methods, such as chemoradiotherapy, thermal radiotherapy, and immunotherapy, can not only enhance the radiotherapy response, but also achieve a synergistic effect.

However, several challenges remain for the development and application of Hf-based cancer therapy. Metal-based nano-enhancers are increasingly trusted as ideal sensitizers (Wardman, 2007), however, NMs containing hafnium elements are not fully biodegradable, which limits their application. Therefore, it is a great need for NPs that are biodegradable, eliminable, or renally cleared. Moreover, hafnium, as a relatively new discovered metal, requires more systematic studies on its biological compatibility and safety to obtain new commercially approved imaging enhancers and therapeutic sensitizers. In future research, multifunctional Hf-NMs will be used to strengthen the combination of radiotherapy and other new-developed cancer therapy, overcoming the limitations of single treatment mode, improve the efficacy of radiotherapy and achieve better synergistic treatment.

In clinical practice, heavy metals such as platinum have been used to treat cancer patients. Platinum-based chemotherapy can disrupt the DNA replication process thus treat cancer, it has been found to be more effective in patients with mutations in the DDR gene pathway, possibly because DDR gene pathway mutations cause the body failing to repair the damage caused by platinum (Basourakos et al., 2017). In reported studies, Hf also induces DNA breaks by enhancing radiotherapy, but whether the properties of Hf itself have therapeutic effects on tumor warrants further investigation, research combining with genomics and proteomics may lead to better precision medicine for cancer patients. Now more nano preparations are explored, their safety will be better guaranteed, more clinical research on Hf NMs can be carried out.

The study of nanomaterials for radioprotection represents another vital application within radiotherapy. Advancements in technology within the field of radiotherapy have been rapid, allowing for the development of increasingly effective treatment plans that better spare the surrounding healthy tissues. One of the focal points of future research lies in harnessing Hf-based NMs to concurrently achieve the dual objectives of sensitizing tumor cells to radiation while shielding normal tissues.

To date, numerous molecular radioprotective drugs have been formulated. For instance, curcumin demonstrates protective capabilities against the detrimental effects of radiation through a free radical scavenging process (Xie et al., 2018). Several studies have developed ROS-scavenging nanomaterials using curcumin in coordination with metal such as ferrum in preclinical models (Malacaria et al., 2021). Coordinating high-Z metals with such radioprotective natural products may simultaneously enhance radiosensitivity and mitigate the side effects of radiotherapy.

We aspire that the ongoing endeavors of researchers and oncologists alike will contribute to the advancement and implementation of Hf-based NMs in the realm of cancer diagnosis and treatment, ultimately leading to a positive impact on patient health.
